# Ingredient Consistency of Commercially Available Polyphenol and Tocopherol Nutraceuticals

**DOI:** 10.3390/pharmaceutics2010050

**Published:** 2010-03-08

**Authors:** Connie M. Remsberg, Renee L. Good, Neal M. Davies

**Affiliations:** Department of Pharmaceutical Sciences, College of Pharmacy, Washington State University, Pullman, WA 99164-6534, USA; E-Mails: connie.remsberg@email.wsu.edu (C.M.R.); renee.good@ucdenver.edu (R.L.G.)

**Keywords:** content analysis, polyphenols, nutraceuticals, vitamin E succinate, pterostilbene, phloretin, myricetin

## Abstract

Label claims of vitamin E succinate and polyphenolic nutraceuticals are assessed. A validated HPLC method was utilized to assess vitamin E succinate products. Three novel LC/MS methods were used to assess the polyphenols, pterostilbene, phloretin, and myricetin, in dietary supplements. The amount of vitamin E succinate varied from 0-130% of the stated label content with two products containing vitamin E acetate rather than vitamin E succinate. Expected polyphenols were found in 7 of the 8 supplement products. None of the polyphenol supplements contained content within 100-120% of label claims. The present study indicates a lack of uniformity in nutraceutical products.

## 1. Introduction

A plethora of nutraceutical products are available and regularly purchased throughout the United States. Unbeknownst to the majority of the population is that the Food and Drug Administration (FDA) considers dietary supplements separate from conventional foods and drug products such as prescription and over-the-counter medications. This results from passage of the Dietary Supplement Health and Education Act of 1994 (DSHEA) under which these products are not subject to rigorous checks on purity and usual quality controls of conventional prescription and non prescription products approved by the FDA [1,2,3]. The nutraceutical manufacturer is responsible for ensuring initial safety of marketed products and only after a product has reached the market does the FDA take action. These post-market responsibilities of the FDA include voluntary adverse effect reporting and monitoring of label claims and accompanying literature [1]. The Federal Trade Commission is responsible for monitoring dietary supplement advertising including false and misleading claims [1]. 

DSHEA was created to assure customer access to dietary supplements. Accordingly, marketing data shows increased sales since the late 1990’s with total sales of nutraceuticals in the billions [2]. Surveys completed by the National Center for Health Statistics (NCHS) further show considerable dietary supplement use by US adults. Data from the National Health and Nutrition Examination Survey (NHANES) indicated 52% of adults had taken a dietary supplement in the past month with 35% taking a multi-vitamin/multi-mineral and 12.7% taking a vitamin E supplement [2]. Another survey by NCHS indicated that in the past 12 months, 17.7% of US adults had used non vitamin/non mineral natural products [4]. 

The extensive use of vitamin E supplements is not surprising. Vitamin E derivatives such as vitamin E succinate have been shown to act as a potent anti-tumor agents that may be helpful in the treatment of Alzheimer’s and other diseases [5,6]. Vitamin E compounds are usually produced and made available in an esterified form as vitamin E acetate (α-tocopheryl acetate) or vitamin E succinate (α-tocopheryl succinate). These esterified forms are generally more stable with respect to storage time and temperature than the unesterified forms. Both the acetate and succinate forms are susceptible to hydrolysis and can be converted to α-tocopherol in the body. Interestingly, the acetate form is rapidly activated within the body, while the activation of the succinate form is slower. Additionally, the succinate form appears to access and benefit areas of the tissues that are not reached by other forms. For this reason, there is a tendency to regard vitamin E succinate as a distinctly different and beneficial compound. Like other supplements, vitamin E products are not under strict regulatory control to ensure quality, stability, and purity.

The recent increase in natural product supplements is also expected as numerous studies have correlated an array of natural product consumption with decreased mortality. Natural product supplements may include herbs (single or mixture), botanical products, or dietary enzymes [4]. A growing subset of natural product supplements are polyphenolic compound isolates and plant extracts known to contain polyphenols. Polyphenols are a class of phytochemicals that are important dietary antioxidants that may possess other potential health benefits including anti-cancer, anti-inflammatory, cardioprotective, anti-fungal, and anti-diabetic activities [7,8,9,10]. Due to these health benefits, a multitude of nutraceuticals claiming to contain these polyphenols have come on to the market. Unfortunately, the quality, stability, purity, and activity of many of these polyphenols are not yet fully understood. In addition, assays to reproducibly measure polyphenol concentrations in either plant material or in nutraceuticals have not been developed or validated. 

Pterostilbene, phloretin, and myricetin are three polyphenolic compounds found in an array of natural sources. Pterostilbene is a structural analogs of resveratrol, a polyphenol with anti-cancer, anti-oxidant, and anti-inflammatory activities [8]. Pterostilbene has shown antioxidant, anti-cancer, anti-diabetic, and other bioactivities [11] and has been isolated from grapes [12,13,14], blueberries [15], and *Pterocarpus marsupium* [16]. Phloretin is a dihydrochalcone polyphenol primary found in apples with antioxidant, antithrombotic properties, and hepatoprotective properties [17,18]. Myricetin, a flavanol polyphenol found in a range of plants including berries, tea, and wine, has shown antioxidant, anticancer, cardioprotective, and anti-diabetic properties [19]. 

In the present study, we assess the content of two separate sets of nutraceutical products. In the first set, we assess the content uniformity of vitamin E succinate supplements; in the second, polyphenolic supplements were assessed for label claims by quantifying pterostilbene, phloretin, and myricetin. We have previously validated a sensitive high-performance liquid chromatography (HPLC) method for the determination of vitamin E succinate [20]. Using our method, vitamin E succinate can be quantified directly without the need for hydrolysis of the succinate moiety [21]. We have additionally validated HPLC methods for quantification of a variety of polyphenols including pterostilbene [22], oxyresveratrol [23], naringenin [24], eriodictyol [25], hesperetin [26], and many others. These methods can be adapted for liquid chromatography/mass spectrometry (LC/MS) use to further increase detection limits. Herein we describe three novel LC/MS methods for the detection and quantification of pterostilbene, phloretin, and myricetin in nutraceutical products. 

## 2. Experimental section

### 2.1. Polyphenol and Vitamin E succinate products

Fourteen preparations containing vitamin E succcinate were selected on their basis of being readily available in United States. Eight polyphenolic nutraceutical products were selected based on claims of containing a polyphenol or claims of containing an extract known to contain a polyphenol. Products analyzed and label claims are reported in Table 1. Lot numbers and expiration dates of the vitamin E products have been previously reported [27]. All lots were within one year of the noted expiry date. Products were purchased on the open market through retail stores, on-line retailers, and direct sales or multi-level marketing companies. Products were not accepted directly from manufacturers. For the vitamin E products, all contained vitamin E succinate between 200-400 IU in each capsule/tablet. It was expected from their labels that all formulations should contain the weight as vitamin E succinate. 

### 2.2. Sample extraction and preparation

Of the vitamin E succinate products, 3 of the 14 preparations were tablets, all the others were capsules. For vitamin E extraction and preparation, 5 tablets or capsules from each container were weighed and transferred to a mortar. They were ground and 3 portions of the resulting powder each equivalent to 10% of the total weight were dissolved in 1 L of water. The resultant was filtered and 1 ml of the filtrate was diluted to 2.5 ml with water. To 0.05 ml of this solution was added internal standard. The mixture was evaporated to dryness and reconstituted in 0.2 ml mobile phase. A 0.1 ml volume of the eluted solution was injected into an isocratic HPLC system.

Of the polyphenol nutraceuticals, 5 of the 8 preparation were capsules, and all others were tablets. For polyphenol extraction and preparation, 1 tablet or the contents of 1 empty capsule were weighed. Each tablet was ground in a mortar to a fine powder. Powders of capsules/tablets were extracted with 4 ml of methanol by placing on a rotating shaker for 3 h. Samples were centrifuged at 2,000 rpm for 5 min. The supernatant was collected and then divided into two groups with 100 µl aliquots for each group. The first group was enzymatically incubated to cleave the glycoside form of the polyphenol into its aglycone form. For this, the 100 µl aliquot was dried to completion under a stream of nitrogen gas and reconstituted in 200 µl phosphate buffer saline (PBS). To this, 20 µl of β-glucosidase from almonds was added and then incubated at 37 °C for 48 h in a hot water bath. Internal standard (25 µl) and 1 ml of cold acetonitrile was added to each sample. Samples were again centrifuged at 2,000 rpm for 5 min and the supernatant dried under a stream of nitrogen gas. The residues were reconstituted with 200 µl mobile phase and 10 µl was injected into the LC/MS system. For the second group, samples were prepared without enzymatic incubation thereby allowing quantification of the aglycone alone. Specifically, to the 100 µl aliquots, 25 µl of internal standard was added. Samples were dried under a stream of nitrogen gas, residues reconstituted in 200 µl mobile phase, and 10 µl was injected into the LC/MC system. By subtracting of the concentration of the non enzymatic incubated sample from the incubated sample, the amount of glycoside can be determined.

**Table 1:** Dietary supplement label claims per capsule/tablet.

**Vitamin E succinate product**	**Label claims of vitamin E succinate per capsule/tablet**				
Bronson® Natural Vitamin E	300 IU				
Puritan’s Pride® Natural “Non Oily” Dry Vitamin E	400 IU				
LifeExtension™ Vitamin E	400 IU				
NOW® Dry Vitamin E	400 IU				
GNC Vitamin E	400 IU				
Jarrow Formulas® Dry Vitamin E	400 IU				
Twinlab® Vitamin E	400 IU				
Carlson® Key-E Vitamin E	400 IU				
Solgar® Natural Vitamin E	400 IU				
Bluebonnet Dry Vitamin E	400 IU				
Allergy Research Group® Vitamin E Succinate	400 IU				
Hypoallergic	400 IU				
American Biologics® Dry Vitamin E Succinate	400 IU				
Super Spectrim® Vitamin E	200 IU				
**Polyphenol product**	**Lot Number**	**Expiration date**	**Label claims per capsule/tablet**		
			**Pterostilbene**	**Phloretin**	**Myricetin**
LifeExtension™ Blueberry extract	27321	12/1/2009	•		†
Source Naturals® Myricetin	723915	11/1/2010			•
Source Naturals® Policosanol Cholesterol Complex	730536	11/1/2010			•
Swanson® Double strength apple cider vinegar	158461	9/1/2009		†	
Swanson® GreenFoods Formulas, Wild blueberry extract	158371	9/1/2009	•		†
Swanson® Superior herbs, Red wine extract	157639	7/1/2009			†
Swanson Ultra® 100% Natural pterostilbene	156251	5/1/2009	•		
Swanson Ultra® Maximum strength apple polyphenols	156948	6/1/2009		†	

† Indicates a product claiming to contain a plant extract known to contain the specific polyphenol.

• Indicates a product claiming an amount of polyphenol.

### 2.3. Analysis methods

To assess content of vitamin E succinate products, a Shimadzu HPLC system (Kyoto, Japan) consisting of an LC-10AT VP pump, a SIL-10AF auto injector, a SPD-M10A VP spectrophotometric diode array detector, and a SCL-10A VP system controller was used. Data collection and integration were accomplished using Shimadzu EZ Start 7.1.1 SP1 software (Kyoto, Japan). The analytical column used was Jones chromatography C_18_ column (3.3 cm × 4.6 mm I.D., 3-µm particle size, Lakewood Colorado USA). The mobile phase consisted of acetonitrile and water (95:5 v/v), filtered and degassed under reduced pressure, prior to use. Separation was carried out isocratically at ambient temperature (25 ± 1 °C), and a flow rate of 1.3 ml/min, with ultraviolet (UV) detection at 205 nm. Standard solutions were prepared in mobile phase. The assay was linear over the range of 1-400 µg/ml with a limit of quantification (LOQ) of 0.001 ug/ml. Vitamin E succinate (TS) and α-tocopheryl acetate standards were purchased from Sigma Chemicals (St. Louis, MO, USA). All analyses were run with quality control samples of known drug concentrations (the analyst was blinded), prepared from the same material as the standards. 

To assess polyphenol content, a liquid chromatographic-electrospray ionization-mass spectrometry (LC-ESI-MS) system was employed. A Shimadzu LCMS-2010 EV liquid chromatograph mass spectrometer system (Kyoto, Japan) connected to the LC portion consisting of two LC-10AD pumps, a SIL-10AD VP auto injector, a SPD-10A VP UV detector, and a SCL-10A VP system controller was used. Data analysis was accomplished using Shimadzu LCMS Solutions Version 3 software. The mass spectrometer conditions consisted of a curved desolvation line (CDL) temperature of 200 °C and a block temperature of 200 °C. The CDL, interface, and detector voltages were -20.0 V, 4.5 kV, and 1.2 kV, respectively. Vacuum was maintained by an Edwards® E2M30 rotary vacuum pump (Edwards, UK). Liquid nitrogen (Washington State University Central Stores) was used as a source of nebulizer gas (1.5 L/min). Each polyphenol was monitored in selected ion monitoring (SIM) negative mode. Standard curves for each compound were linear over the concentration ranges of 0.05-100 µg/ml. The LOQ was 50 ng/ml for each compound. *Trans*-pterostilbene and pinosylvin standards were purchased from Sequoia Research Products Ltd. (Oxford, United Kingdom). Phloretin, daidzein, and myricetin standards were purchased from Sigma Chemicals (St. Louis, Mo, USA).

To assess pterostilbene content, a previously validated HPLC method was adapted for LC/MS use [22]. The analytical column used was a Phenomenex C_18_ (250 × 4.60 mm, 5-µm). The mobile phase consisted of acetonitrile and HPLC water (50:50, v/v) at a flow rate of 1 ml/min. Pinosylvin was used as an internal standard. Pterostilbene was monitored in selected ion monitoring (SIM) negative mode with the single plot transition at m/z 255; pinosylvin at m/z 211.

For assessment of phloretin content, a novel LC/MS method was developed using a Chiralcel OD-RH column (150 mm × 4.6 mm i.d., 5-µm). The mobile phase consisted of acetonitrile, water and formic acid (30:70:0.08, v/v/v) at a flow rate of 0.4 ml/min. Daidzein was used as an internal standard. Phloretin and daidzein were monitored in SIM negative mode with the single plot transitions at m/z 273 and 253, respectively.

To quantify myricetin, a third LC/MS method was developed. Separation was achieved using a Phenomenex C_18_ (250 × 4.60 mm, 5-µm). The mobile phase consisted of acetonitrile, water, and formic acid (30:70:0.01, v/v/v) at a flow rate of 0.4 ml/min. Daidzein was used as an internal standard. Myricetin was monitored in SIM negative mode with the single plot transitions at m/z 317.

## 3. Results and discussion

### 3.1. Vitamin E succinate products

The previously validated HPLC method was successfully applied to the detection and quantification of vitamin E succinate in dietary supplements. The results of the analysis are reported in Table 2. Appropriate labeling was defined as a product that contained a least 100% and no more than 120% of its claimed amount of vitamin E succinate (taking into account a margin of error of five percent for the HPLC method). In addition, if claimed to contain vitamin E succinate the products had to contain no vitamin E acetate form. The amount of vitamin E succinate in the product also had to be clearly and accurately stated in the labeling. A pass was based on the meeting the above criteria. We should note that the current criteria used by the United States Pharmacopeia (USP) for vitamin E products is 90-165% of labeled amount. After analysis, two products did not pass our aforementioned criteria. In addition, many products also were found to contain small amounts of gamma-tocopheryl succinate (less than 1%).

**Table 2:** Vitamin E succinate content in assessed supplements expressed as a percent of label claims in random order (mean ± SD). Two products contained vitamin E acetate while many products included gamma-tocopheryl succinate.

Number	Vitamin E succinate (%)	Vitamin E acetate (%)	Gamma- tocopheryl succinate(%)
1	112.5 ± 8.4	0	0.38
2	116.9 ± 8.3	0	0.46
3	104.2 ± 9.7	0	0.71
4	103.9 ± 3.3	0	0.47
5	99.5 ± 7.1	0	0.37
6	106.1 ± 2.8	0	0.29
7	110.5 ± 5.9	0	0.74
8	106.4 ± 5.5	0	0.41
9	116.8 ± 4.2	0	0.94
10	110.0 ± 5.1	0	0.55
11	103.7 ± 2.9	0	0.81
12	119.3 ± 4.3	0	0.57
13	0	9.3	0
14	0	84.7	0

In usual doses, vitamin E succinate is a nontoxic substance and its use is unlikely to be associated with serious side effects even when relatively large amounts are ingested. However, the efficacy of the drug may depend on the amount ingested as vitamin E succinate. As it stands from our analysis the amount of vitamin E succinate stated on product labels may not be an accurate reflection of the active ingredient contained for some products. This inconsistency may account for variability and differences in results using different vitamin E succinate preparations in clinical trials. Similar results to ours have been reported by Feifer *et al.* Their work indicated a percent difference between measured and stated dose for vitamin E products that ranged from -41% to +57% [28]. If our criteria for assessment are employed, only 2 of the 7 examined products contained accurate claims. 

Neither the United States government nor any other agency is routinely testing vitamin E succinate supplements for their content or quality. The lack of this basic pharmaceutic information of vitamin E succinate supplements is in part due to a deficit of simple validated and direct methods of analysis of vitamin E succinate available in the literature. The HPLC method used here was successfully applied to the content evaluation of vitamin E succinate products.

### 3.2. Polyphenol nutraceuticals

Three novel LC/MS assays were successfully applied to the quantification of pterostilbene, phloretin, and myricetin in nutraceutical products as shown in Table 3. When assessing content, both the aglycone and glycoside forms were measured. Previous reports have indicated that most polyphenols exist as glycosides with a smaller amount existing as the aglycone or parent compound. It is hypothesized that the glycoside form is cleaved in the gastrointestinal tract to the more bioactive aglycone [24]. In the present study, polyphenols were found in both the glycoside and aglycone forms.

**Table 3:** Polyphenol content in selected nutraceutical products in random order (mg per tablet/capsule). If a specific polyphenol amount was claimed, the amount in mg is indicated below.

Number	Pterostilbene			Phloretin			Myricetin		
	Claimed	Aglycone	Glycoside	Claimed	Aglycone	Glycoside	Claimed	Aglycone	Glycoside
1		0	0		0.0024	0	10 mg	2.65	0.132
2	0.1 mg	0.232	0		0.0050	0.0048	†	0.150	0.062
3		0	0		0.0019	0	100 mg	2.21	0.012
4		0	0	†	0.105	0		0	0
5	0.25 mg	0.552	0		0	0	†	0.035	0.0674
6	30 mg	1.28	0.186		0	0		0	0
7		0	0		0.0059	0	†	0.030	0.0014
8		0	0	†	0	0		0	0

† Indicates a product claiming to contain a plant extract known to contain the specific polyphenol.

To assess label claims of polyphenol, appropriate labeling was defined as a product that contained at least 100% and no more than 120% of its claimed amount. If the label claimed an extract known to contain a polyphenol, appropriate labeling was attained if any amount of the polyphenol was quantifiable. Of the products claiming a particular polyphenol or plant extract, 7 of the 8 products contained the stated polyphenol constituent; however, the polyphenol amounts assessed did not approach stated label claims. In fact, label claims using the aforementioned criteria were not attained in any of the products examined. Percentages of detected amounts varied from 2% to 230% of label claims but were not within the range of 100% to 120%. Several products contained polyphenols that were not expressed on label claims.

The present study indicates a lack of uniformity in botanical dietary supplements. This agrees with the studies of other research groups investigating label accuracy of polyphenol-rich soy isoflavonoids [29] and green tea extracts [30] as well as hypericin content in St. John’s Wort products [31]. Examination of 13 soy isoflavone products by Chua *et al.* indicated inadequate levels of isoflavones with only 4 of the 13 products containing >90% of the claimed content [29]. In a study by Seeram *et al.*, 19 commercially available green tea dietary supplements were assessed for catechin and total polyphenol content. Eleven products with label claims of epigallocatechin gallate (EGCG) were assessed with EGCG levels ranging from 12 to 143% [30]. Of the 11 products, only three products met passing criteria set in the study [30]. Five of the 19 products made claims on total polyphenol content with assessed levels ranging from 14 to 36% of label claims [30]. No products contained the claimed amount of total polyphenols [30]. Finally, studies by Glisson *et al.* on hypericin content in St. John’s Wort products indicated that none of the 13 products examined contained between 90-110% of stated label claim [31].

The lack of uniformity described here and in other studies is not surprising as there are no standards for testing botanical dietary supplements, nor is there a government agency that evaluates the content of these botanical products. This lack of analysis is also likely in part due to a limited number of validated analysis techniques such as HPLC and LC/MS detection assays. Here we evaluate three LC/MS methods for measuring the polyphenol content of selected nutraceutical products. Not only were these methods successfully able to assess claims of a set amount of polyphenol, but they were also used to assess claims of particular plant extracts known to contain a particular polyphenol. These applied methods indicate a lack in uniformity of these botanical products, making further assessment of polyphenolic content uniformity and standardization of nutraceutical products and claims warranted.

The developed methods have potential to assess the content of a wide range of nutraceuticals. The detection methods for pterostilbene, phloretin, and myricetin can be used to evaluate label claims of these polyphenols. Additionally, the developed detection methods have potential for evaluating plant extract claims. For instance, pterostilbene can be a marker of blueberry, grape, and *Pterocarpus marsupium* extracts; phloretin for apple extracts; and myricetin for berries, tea, and wine extracts. From these results, we propose that measuring polyphenol content may provide an appropriate means to monitor botanical product claims and their content uniformity.

## 4. Conclusions

Herein, we present the analysis of vitamin E succinate products using a previously validated HPLC method as well as the analysis of polyphenolic botanical supplements using three LC/MS methods for detection of pterostilbene, phloretin, and myricetin. Results indicate that 12 of the 14 evaluated vitamin E products passed evaluation criteria with vitamin E succinate claims within 100-120% of measured levels. Analysis of polyphenol content indicated presence of expected polyphenol in seven of the eight products; however, none of the polyphenol supplements passed strict evaluation criteria of content with 100-120% of label claims. 

It is therefore apparent from this study as well as analysis of other nutraceutical products [21,32,33,34] that variability exists between products and manufacturers. This lack of uniformity may be occurring in vitamin, mineral, or botanical supplements. The lack of regulation likely results in part from a deficit of detection methods that can allow reasonable analysis and quantification. Studies such as this one may allow development of new methods that can be easily applied to the assessment of nutraceutical products and label claims. Given the powerful pharmacological effects and health-benefits of these products it is recommended that nutraceuticals be correctly and accurately labeled and regulatory procedures be developed for standardizing manufacturing and content uniformity of these nutraceutical supplements.

## References

[1] Dietary Supplements. U.S. Food and Drug Administration. http://www.fda.gov/food/DietarySupplements/default.htm.

[2] Radimer K., Bindewald B., Hughes J., Ervin B., Swanson C., Picciano M.F. (2004). Dietary supplement use by US adults: data from the National Health and Nutrition Examination Survey, 1999-2000. Am. J. Epidemiol..

[3] Radimer K.L., Subar A.F., Thompson F.E. (2000). Nonvitamin, nonmineral dietary supplements: issues and findings from NHANES III. J. Am. Diet Assoc..

[4] Barnes P.M., Bloom B., Nahin R. (2008). Complementary and alternative medicine use among adults and children: United States, 2007. Natl. Health Stat. Report..

[5] Fariss M.W., Nicholls-Grzemski F.A., Tirmenstein M.A., Zhang J.G. (2001). Enhanced antioxidant and cytoprotective abilities of vitamin E succinate is associated with a rapid uptake advantage in rat hepatocytes and mitochondria. Free Radic. Biol. Med..

[6] Fariss M.W., Zhang J.G. (2003). Vitamin E therapy in Parkinson's disease. Toxicology.

[7] Fraga C.G. (2007). Plant polyphenols: how to translate their *in vitro* antioxidant actions to *in vivo* conditions. IUBMB Life.

[8] Fremont L. (2000). Biological effects of resveratrol. Life Sci..

[9] Giovannini C., Scazzocchio B., Vari R., Santangelo C., D'Archivio M., Masella R. (2007). Apoptosis in cancer and atherosclerosis: polyphenol activities. Ann. Ist. Super. Sanita.

[10] Roupe K., Remsberg C., Yanez J., Davies N. (2006). Pharmacometrics of Stilbenes: Seguing Towards the Clinic. Curr Clin Pharmacol.

[11] Remsberg C.M., Yanez J.A., Ohgami Y., Vega-Villa K.R., Rimando A.M., Davies N.M. (2008). Pharmacometrics of pterostilbene: preclinical pharmacokinetics and metabolism, anticancer, antiinflammatory, antioxidant and analgesic activity. Phytother Res.

[12] Pezet R., Pont V.  (1988). Mise en evidence de pterostilbene dans les grappes de Vitis vinifera (var. Gamay et Pinot). Plant Physiol. Biochem..

[13] Douillet-Breuil A.C., Jeandet P., Adrian M., Bessis R. (1999). Changes in the phytoalexin content of various Vitis spp. in response to ultraviolet C elicitation. J. Agric. Food Chem..

[14] Adrian M., Jeandet P., Douillet-Breuil A.C., Tesson L., Bessis R. (2000). Stilbene content of mature Vitis vinifera berries in response to UV-C elicitation. J. Agric. Food Chem..

[15] Rimando A.M., Kalt W., Magee J.B., Dewey J., Ballington J.R. (2004). Resveratrol, pterostilbene, and piceatannol in vaccinium berries. J. Agric. Food Chem..

[16] Manickam M., Ramanathan M., Jahromi M.A., Chansouria J.P., Ray A.B. (1997). Antihyperglycemic activity of phenolics from Pterocarpus marsupium. J. Nat. Prod..

[17] An R.B., Park E.J., Jeong G.S., Sohn D.H., Kim Y.C. (2007). Cytoprotective constituent of Hoveniae Lignum on both Hep G2 cells and rat primary hepatocytes. Arch. Pharm. Res..

[18] Stangl V., Lorenz M., Ludwig A., Grimbo N., Guether C., Sanad W., Ziemer S., Martus P., Baumann G., Stangl K. (2005). The flavonoid phloretin suppresses stimulated expression of endothelial adhesion molecules and reduces activation of human platelets. J. Nutr..

[19] Ong K.C., Khoo H.E. (1997). Biological effects of myricetin. Gen. Pharmacol..

[20] Good R.L., Roupe K.A., Fukuda C., Clifton G.D., Fariss M.W., Davies N.M. (2005). Direct high-performance liquid chromatographic analysis of D-tocopheryl acid succinate and derivatives. J. Pharm. Biomed. Anal..

[21] Russell A.S., Aghazadeh-Habashi A., Jamali F. (2002). Active ingredient consistency of commercially available glucosamine sulfate products. J. Rheumatol..

[22] Remsberg C.M., Yanez J.A., Roupe K.A., Davies N.M. (2007). High-performance liquid chromatographic analysis of pterostilbene in biological fluids using fluorescence detection. J. Pharm. Biomed. Anal..

[23] Bertram R.M., Takemoto J.K., Remsberg C.M., Vega-Villa K.R., Sablani S., Davies N.M. (2009). High-performance liquid chromatographic analysis: applications to nutraceutical content and urinary disposition of oxyresveratrol in rats. Biomed. Chromatogr..

[24] Yanez J.A., Davies N.M. (2005). Stereospecific high-performance liquid chromatographic analysis of naringenin in urine. J. Pharm. Biomed. Anal..

[25] Yanez J.A., Miranda N.D., Remsberg C.M., Ohgami Y., Davies N.M. (2007). Stereospecific high-performance liquid chromatographic analysis of eriodictyol in urine. J Pharm Biomed Anal.

[26] Yanez J.A., Teng X.W., Roupe K.A., Davies N.M. (2005). Stereospecific high-performance liquid chromatographic analysis of hesperetin in biological matrices. J Pharm Biomed Anal.

[27] Woody R., Fukuda C., Roupe K., Fariss M., Clifton G., Davies N. Alpha-tocopherol Succinate: Analysis, Content Uniformity, Pharmacokinetics, Tissue Distribution and Anti-Cancer Activity. Canadian Society of Pharmaceutical Sciences (CSPS) annual meeting.

[28] Feifer A.H., Fleshner N.E., Klotz L. (2002). Analytical accuracy and reliability of commonly used nutritional supplements in prostate disease. J. Urol..

[29] Chua R., Anderson K., Chen J., Hu M. (2004). Quality, labeling accuracy, and cost comparison of purified soy isoflavonoid products. J. Altern. Complement. Med..

[30] Seeram N.P., Henning S.M., Niu Y., Lee R., Scheuller H.S., Heber D. (2006). Catechin and caffeine content of green tea dietary supplements and correlation with antioxidant capacity. J. Agric. Food Chem..

[31] Glisson J.K., Rogers H.E., Abourashed E.A., Ogletree R., Hufford C.D., Khan I. (2003). Clinic at the health food store? Employee recommendations and product analysis. Pharmacotherapy.

[32] Draves A.H., Walker S.E. (2003). Analysis of the hypericin and pseudohypericin content of commercially available St John's Wort preparations. Can. J. Clin. Pharmacol..

[33] Green G.A., Catlin D.H., Starcevic B. (2001). Analysis of over-the-counter dietary supplements. Clin. J. Sport Med..

[34] Nelson M.H., Cobb S.E., Shelton J. (2002). Variations in parthenolide content and daily dose of feverfew products. Am. J. Health Syst. Pharm..

